# Alterations of Thalamic Nuclei Volumes and the Intrinsic Thalamic Structural Network in Patients with Multiple Sclerosis-Related Fatigue

**DOI:** 10.3390/brainsci12111538

**Published:** 2022-11-13

**Authors:** Yujing Li, Jun Wang, Tingli Yang, Pengfei Zhang, Kai Ai, Min Li, Rui Wang, Xinying Ren, Diaohan Xiong, Guangyao Liu, Na Han, Tiejun Gan, Jing Zhang

**Affiliations:** 1 Second Clinical School, Lanzhou University, Lanzhou 730030, China; 2 Department of Magnetic Resonance, Lanzhou University Second Hospital, Lanzhou 730030, China; 3 Gansu Province Clinical Research Center for Functional and Molecular Imaging, Lanzhou 730030, China; 4 Department of Clinical and Technical Support, Philips Healthcare, Xi’an 710000, China

**Keywords:** multiple sclerosis, magnetic resonance imaging, thalamus, volume, network

## Abstract

Fatigue is a debilitating and prevalent symptom of multiple sclerosis (MS). The thalamus is atrophied at an earlier stage of MS and although the role of the thalamus in the pathophysiology of MS-related fatigue has been reported, there have been few studies on intra-thalamic changes. We investigated the alterations of thalamic nuclei volumes and the intrinsic thalamic network in people with MS presenting fatigue (F-MS). The network metrics comprised the clustering coefficient (*Cp*), characteristic path length (*Lp*), small-world index (*σ*), local efficiency (*E_loc_*), global efficiency (*E_glob_*), and nodal metrics. Volumetric analysis revealed that the right anteroventral, right central lateral, right lateral geniculate, right pulvinar anterior, left pulvinar medial, and left pulvinar inferior nuclei were atrophied only in the F-MS group. Furthermore, the F-MS group had significantly increased *Lp* compared to people with MS not presenting fatigue (NF-MS) (2.9674 vs. 2.4411, *P^AUC^* = 0.038). The F-MS group had significantly decreased nodal efficiency and betweenness centrality of the right mediodorsal medial magnocellular nucleus than the NF-MS group (false discovery rate corrected *p* < 0.05). The F-MS patients exhibited more atrophied thalamic nuclei, poorer network global functional integration, and disrupted right mediodorsal medial magnocellular nuclei interconnectivity with other nuclei. These findings might aid the elucidation of the underlying pathogenesis of MS-related fatigue.

## 1. Introduction

Fatigue is a debilitating and prevalent symptom of multiple sclerosis (MS) that exerts a negative socioeconomic effect and reduces work productivity. Between 76% and 97% of patients with MS (PwMS) list fatigue as a symptom [1], with up to 40% of patients reporting it as their most disabling symptom [2]. Moreover, approximately 50% of patients develop cognitive impairments [3]. Although fatigue can be linked to disease mechanisms, such as inflammation and demyelination [4], and other clinical aspects of MS, such as sleep disorders [5], MS-related fatigue is primarily central with cognitive, physical, and psychosocial elements [6]. The term “central fatigue” was proposed by Chaudhuri and Behan in 2000 and primarily refers to the inability to execute or sustain both mental (cognitive) and physical (motor) tasks that require self-motivation [7]. PwMS can experience chronic central fatigue (lasting ≥6 months) as opposed to the short-term physiological fatigue experienced by healthy people [8]. Although several pathogenic substrates have been postulated to explain the formation of primarily central fatigue in PwMS, its etiology remains obscure. The potential causes include disruptions in the cortico-subcortical connections involving the frontal cortex, thalamus, and basal ganglia [9].

Despite a substantial amount of evidence supporting its involvement, few studies focused on the thalamus as compared to the basal ganglia in efforts to describe the pathophysiology of MS-related fatigue. A structural analysis of the subcortical nuclei revealed that PwMS with high fatigue had significantly reduced thalamic volume [10]. A diffusion tensor imaging study that detected microstructural changes in the cerebrum reported that PwMS with fatigue but normal cognitive tests exhibited reduced thalamic fractional anisotropy (FA) and increased mean diffusivity [11]. A combined positron emission computed tomography/magnetic resonance imaging (MRI) study demonstrated that the total fatigue score negatively correlated with bilateral thalami atrophy and subsequently with the resting cerebral glucose metabolic rate [12]. Increased T1 relaxation times and decreased cerebral blood flow in the thalamus were also correlated with fatigue [13]. All these studies supported the pivotal role of the thalamus in the pathophysiology underlying MS-related fatigue. The thalamus is an inhomogeneous structure and can be divided into various nuclei, each with a unique structure, and has widespread reciprocal connections to the cortical and subcortical regions. Therefore, the thalamus is important as an information relay and integration center, and its involvement has been associated with other clinical symptoms in PwMS, including depression, pain, and cognitive impairment. A study that assessed the relationship between increased atrophy and worsened depressive symptom over time revealed that the left mediodorsal nucleus of the thalamus in which atrophy progressed was associated with increased depressive symptom severity [14]. Cognitive dysfunctions are widely demonstrated in PwMS and many recent studies confirmed their link with thalamic atrophy [15,16]. For example, Rojas et al. observed a significantly decreased thalamus volume in patients with relapsing–remitting MS (PwRRMS) who developed cognitive impairment after two years of follow-up after controlling for the influence of global and neocortex atrophy [17]. In PwMS, central neuropathic pain was associated with impaired spinothalamic-thalamocortical pathways [18]. However, no studies have investigated the alterations of individual thalamic nuclei volumes in PwMS presenting fatigue (F-MS).

Connectome analysis involves both structural and functional connectivity and has recently gained prominence as a formal framework for network research, which resulted in a paradigm change in various neurological diseases [19,20,21]. Graph theory is the most commonly utilized mathematical tool in connection investigations for analyzing and quantifying brain networks [22]. The connectivity properties of motor and sensory cortical districts in PwRRMS were studied using graph theoretical analysis [23]. The main result of that electroencephalography study was that functional connectivity changes of the left sensory cortical network at rest occurred in F-MS mediated by beta band oscillatory activity. However, to the best of our knowledge, no study has investigated the intrinsic thalamic network changes, especially based on F-MS.

This research investigated the alterations of thalamic nuclei volumes and the intrinsic thalamic network in PwRRMS, which may be critical in the pathophysiology of MS-related fatigue. We anticipated that the aforementioned characteristics differed between the F-MS and PwMS not presenting fatigue (NF-MS) subtypes.

## 2. Materials and Methods

### 2.1. Study Population

Fifty patients diagnosed with clinically definite RRMS based on the 2017 revised McDonald criteria [24] were recruited from Lanzhou University Second Hospital. Patients with thalamic demyelination were excluded to eliminate the effect of thalamic lesions on thalamic atrophy. The Expanded Disability Status Scale (EDSS) was used to measure disease severity and retain patients with limited disability. All participants were also tested with the Montreal Cognitive Assessment (MoCA), the Beck Depression Inventory II (BDI-II), and the Pittsburgh Sleep Quality Index (PSQI) to reduce potential result confounding by cognitive impairment, depressive symptoms, or sleep quality. The inclusion criteria were: (1) age between 18 and 45 years; (2) no clinical relapse and lack of new or enlarged (i.e., active) T2 lesions for at least 3 months; (3) stable disease-modifying treatment and no corticosteroid therapy for at least 3 months; (4) EDSS score of up to 4; (5) MoCA score > 26; (6) BDI-II score < 14; and (7) no sleep disorders. MS-related fatigue was assessed using the self-reported fatigue severity scale (FSS), which consists of nine items on a 7-point scale and focuses on physical fatigue. Patients were considered to have significant fatigue if they had a mean FSS score of ≥4 [25]. Accordingly, the patients were divided into F-MS (n = 25) and NF-MS groups (n = 25). We also enrolled 40 healthy controls (HCs) age-, sex-, and education-matched to the patients. All HCs had normal neurological findings and brain MRI. The Second Hospital of Lanzhou University Ethics Committee approved the project (scientific research project ethics approval number: 2021A-526). All participants provided written informed consent.

### 2.2. MRI Acquisition

Brain MRI data were collected using a 3.0 T MR scanner (Ingenia CX, Philips Healthcare, Best, The Netherlands) with a 32-channel phased-array head coil. The following structural sequences were acquired: (1) sagittal T1-weighted 3D magnetization prepared rapid acquisition gradient echo (MPRAGE) sequence (repetition time [TR] = 7.9 ms; echo time [TE] = 3.5 ms; flip angle = 8°, matrix = 256 × 256 × 360, voxel size = 1 × 1 × 1 mm), and (2) sagittal T2 fluid-attenuated inversion recovery (FLAIR) 3D sequence (TR = 4800 ms; TE = 340 ms; flip angle = 180°, matrix = 252 × 251 × 260, voxel size = 1 × 1 × 1 mm).

### 2.3. Cerebrospinal Fluid (CSF) Examination

CSF (5 mL) was collected at disease diagnosis from all participating patients and immediately sent to the laboratory. The CSF was examined according to the recommendations of the Italian Association for Neuroimmunology [26]. Oligoclonal band (OB) positivity was defined by the presence of at least two bands in the CSF but none in the plasma at the same time point.

### 2.4. Automatic Lesion Segmentation and Filling

In the patients, T2 hyperintense white matter (WM) lesions on the FLAIR images were automatically segmented and quantified using the lesion growth algorithm [27] in the Lesion Segmentation Tool (LST) for Statistical Parametric Mapping (SPM12, London, UK). To improve the tissue segmentation step in FreeSurfer preprocessing for the thalamic nuclei volume assessments, T1 hypointense lesions at the corresponding anatomical locations were identified and filled using the filling algorithm in the same toolbox.

### 2.5. Thalamic Nuclei Volume Analysis

**Figure 1:**
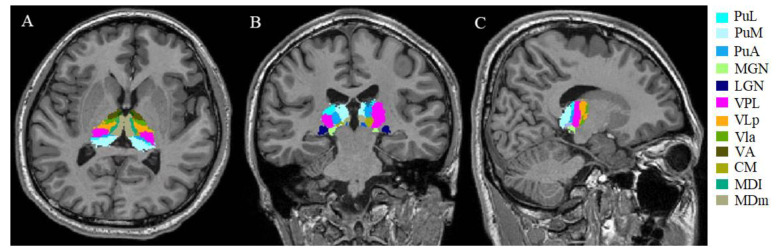
Example of segmentation of thalamic nuclei. Segmentation and labeling of thalamic nuclei in axial plane (**A**), coronal plane (**B**), and sagittal plane (**C**) generated by FreeSurfer (not all segmentations are shown). The segmentations are overlaid on the 3D T1-weighted scan. PuL: pulvinar lateral, PuM: pulvinar medial, PuA: pulvinar anterior, MGN: medial geniculate, LGN: lateral geniculate, VPL: ventral posterolateral, VLp ventral lateral posterior, VLa: ventral lateral anterior, VA: ventral anterior, CM: centromedian, MDl: mediodorsal lateral parvocellular, MDm: mediodorsal medial magnocellular nucleus.

Volumetric analysis of lesion-filled 3D T1-weighted images was performed using the recon-all function in FreeSurfer software (version 7.2.0, Boston, MA, USA). Each image was carefully inspected in the sub-step, which included Talairach transform computation, skull stripping, and WM segmentation. The T1 hypointense lesions that were not effectively filled using the LST tool were manually filled with neighboring normal WM values using the TkMedit function. Other errors were manually corrected and reprocessed (e.g., the addition of control points in incorrect gray matter/WM boundary segmentation). Next, individual thalamic nuclei were automatically segmented using the segmentThalamicNuclei.sh function, which divided each thalamic hemisphere into 25 regions (Figure 1) [28]. A visual inspection was performed using the freeview function to determine whether the thalamic nuclei segmentation had been conducted properly. Considering the absence of errors in this segmentation, all participants were retained in the group analysis. With the above steps, we obtained the participants’ total intracranial volumes and absolute individual thalamic nuclei volumes. The volumetric measures were calculated using the following equation: structural volume (%) = (absolute structural volume/estimated total intracranial volume) × 100 [15].

### 2.6. Graph Theoretical Analysis of the Intrinsic Thalamic Network

Intrinsic thalamic structural network was performed using MATLAB code in the Brain Connectivity Toolbox (MATLAB 2018b, Natick, MA, USA). The thalamic nuclei, which had been divided into 25 subregions in each hemisphere, acted as nodes of the unweighted and undirected network. First, a structurally weighted connectivity matrix Cij (i, j = 1, 2,…, N; here, N = 50) was generated for each group by calculating the partial correlation coefficients across individuals between the volumes of every pair of thalamic subregions. Before the correlation analysis, a linear regression was carried out on every subregion to control the effects of age, sex, and lesion load. The resulting residuals were used to substitute the raw thalamic nucleus volume values. Subsequently, the partial correlation matrix Cij of each group was thresholded into a simpler binarized matrix Aij = [aij], where aij was set to 1 if the absolute value of the correlation Cij between thalamic subregions i and j was larger than a given correlation threshold; otherwise, it was set to 0. The sparsity threshold was identified as 0.05–0.40 in 0.01 increments [29]. In this range, the matrix Cij was thresholded repeatedly and calculated both global and regional network properties at each value. The global metrics in the intrinsic thalamic structural network comprised the clustering coefficient (*Cp*), characteristic path length (*Lp*), normalized clustering coefficient (*γ*), normalized characteristic path length (*λ*), small-world index (*σ*), local efficiency (*E_loc_*), and global efficiency (*E_glob_*). The *σ* was calculated by dividing *γ* by *λ*, obtained from averaging > 150 randomized networks of the same size and degree distribution. The regional metrics included the nodal degree, nodal efficiency, and nodal betweenness. We also calculated the area under the curve (AUC) for each metric, which yielded a summarized scalar for topological network characterization independent of single threshold selection.

### 2.7. Statistical Analysis

The data normality was assessed using the Shapiro–Wilk test and visual histogram inspection. Depending on the data distribution and variable type, sample characteristics between the F-MS and NF-MS groups and between the F-MS, NF-MS, and HCs groups were compared using Pearson’s chi-square test, the Mann–Whitney U test, and analysis of variance (ANOVA) with post hoc Bonferroni’s test. The *p*-value the ANOVA were corrected for multiple hypotheses testing using the false discovery rate (FDR) method (*p* < 0.05). The thalamic nuclei volume differences among the three groups were compared using the ANOVA with post hoc Bonferroni’s test and the *p*-value was set at 0.001 (0.05/50 = 0.001, Bonferroni corrections). Correlation analysis was measured by the Pearson correlation test or Spearman rank correlation test and the *p*-value was also set at 0.001. Furthermore, we performed nonparametric permutation tests with 5000 permutations on the AUC of each network metric to assess the between-group statistical significance of the differences. Regarding the regional metrics, the FDR method was used as the multiple comparison correction (*p* < 0.05). All statistical tests were performed using MATLAB and IBM SPSS Statistics software (version 26.0, Armonk, NY, USA).

## 3. Results

### 3.1. Demographic and Clinical Characteristics

Table 1 summarizes the main demographic, clinical, and conventional MRI characteristics of the final data set of the F-MS (n = 25), NF-MS (n = 25), and HC (n = 40) groups. The sex distribution was not different between the three groups. The proportion of patients with IgG OB in the CSF did not differ in the F-MS and NF-MS groups. The FSS scores were significantly higher in the F-MS group compared to both the HC and NF-MS groups (both, *p* < 0.001), while the other clinical scores were not significantly different. Compared to the HCs, the F-MS subtype exhibited subcortical gray matter atrophy (*p* = 0.002), whereas the NF-MS subtype exhibited subcortical gray matter (*p* = 0.001) and WM atrophy (*p* = 0.035). No differences were detected for the cortex volume, subcortical gray matter volume, total gray matter volume, WM volume, or estimated total intracranial volume between the F-MS and NF-MS groups.

### 3.2. Thalamic Nuclei Volumes

**Figure 2:**
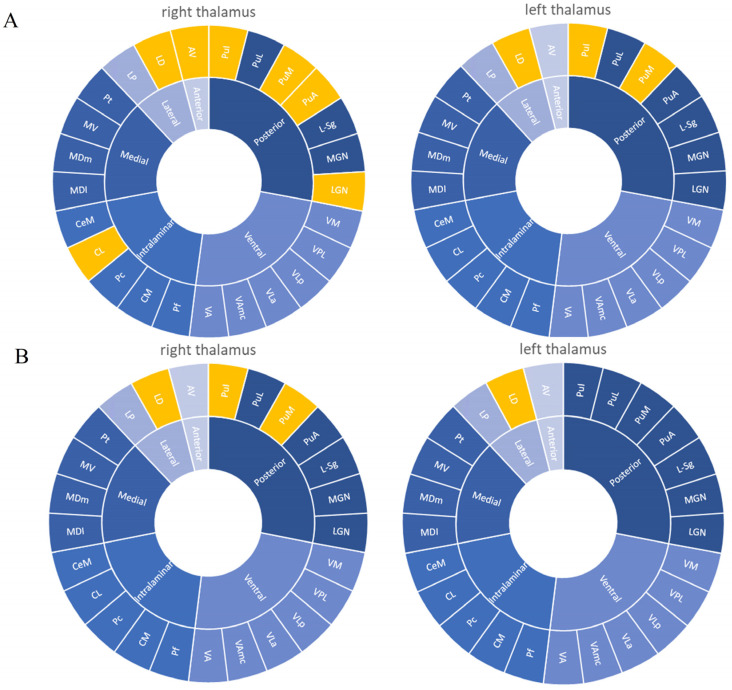
Significant differences of volume changes in individual thalamic nuclei according to presence or absence of fatigue symptom in the patients. (**A**) F-MS vs. HC results. (**B**) NF-MS vs. HC results. Yellow regions indicate decreased volumes in PwMS compared to HCs. In the F-MS group, the right anteroventral, bilateral laterodorsal, right central lateral, right lateral geniculate, right pulvinar anterior, bilateral pulvinar medial, and bilateral pulvinar inferior nuclei volumes were decreased compared to that of the HCs (**A**). In the NF-MS group, the bilateral laterodorsal, right pulvinar medial, and right pulvinar inferior nuclei volumes were decreased compared to that of the HCs (**B**). AV: Anteroventral, LD: laterodorsal, LP: lateral posterior, VA: ventral anterior, VAmc: ventral anterior magnocellular, VLa: ventral lateral anterior, VLp: ventral lateral posterior, VPL: ventral posterolateral, VM: ventromedial, CeM: central medial, CL: central lateral, Pc: paracentral, CM: centromedian, Pf: parafascicular, Pt: paratenial, MV: medial ventral, MDm: mediodorsal medial magnocellular, MDl: mediodorsal lateral parvocellular, LGN: lateral geniculate, MGN: medial geniculate, L-Sg: suprageniculate, PuA: pulvinar anterior, PuM: pulvinar medial, PuL: pulvinar lateral, Pul: pulvinar inferior.

Table 2 reveals the differences in the whole thalamus volumes and individual thalamic nuclei volumes among the F-MS, NF-MS, and HC groups. There were no significant differences in the total volume of the thalamus between the F-MS, NF-MS, and HC groups. Overall, the F-MS group had significantly lower left thalamic volume than the HC group (0.5049% vs. 0.5687%, *p* = 0.0157), and the left thalamic volume of the NF-MS group had a tendency to be lower than that of the HC group (0.5284% vs. 0.5687%, *p* = 0.1426). Overall, the right thalamic volume was significantly lower in both the F-MS (0.4588% vs. 0.5232%, *p* = 0.0047) and NF-MS groups (0.4583% vs. 0.5232%, *p* = 0.0067) than in the HC group. The whole thalamus volume in the right and left hemispheres was not significantly different between the F-MS and NF-MS groups. Individual thalamic nucleus volumetric differences were observed for each MS subtype when compared to the HC group (Figure 2). The bilateral laterodorsal, right pulvinar medial, and right pulvinar inferior nuclei volumes were decreased in both the F-MS and NF-MS groups. However, the right anteroventral, right central lateral, right lateral geniculate, right pulvinar anterior, left pulvinar medial, and left pulvinar inferior nuclei volumes were decreased only in the F-MS group. Differences in thalamic nucleus volume between F-MS and NF-MS groups were not observed.

### 3.3. Intrinsic Thalamic Global Network

All patients and HCs exhibited a small-world architecture at all connection densities. Table 3 displays the differences in the average values and AUCs of the intrinsic thalamic global metrics in the three groups. In the F-MS subtype, the *Lp* was significantly increased compared to that in the HCs (2.9883 vs. 2.4339, *P^AUC^* = 0.013). In the F-MS and NF-MS subtypes, only *Lp* was considerably different (2.9674 vs. 2.4411, *P^AUC^* = 0.038). The global network properties (*Lp*, *E_glob_, E_loc_*, *Cp*, and *σ*) of the NF-MS subtypes were not significantly different from that of the HCs.

### 3.4. Intrinsic Thalamic Regional Network

**Figure 3:**
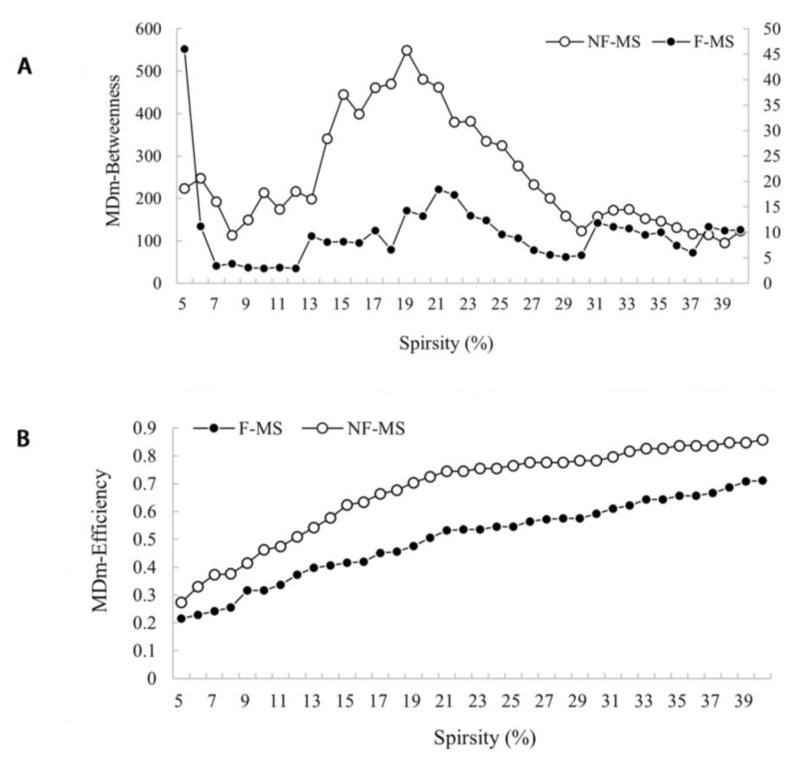
Nodal betweenness centrality (**A**) and nodal efficiency (**B**) for a range of sparsity in the F-MS (●) and NF-MS (○) groups. Data were analyzed by comparing the AUCs among the parameters for each group. The FDR was used to correct for multiple comparison. Relative to NF-MS group, F-MS group exhibited reduced nodal betweenness centrality (**A**) and nodal efficiency (**B**) in the right mediodorsal medial magnocellular nuclei (all with FDR corrected *p* < 0.05). F-MS: Patients with multiple sclerosis presenting fatigue; NF-MS: Patients with multiple sclerosis not presenting fatigue; HCs: Healthy controls.

The nodal degree centrality, betweenness centrality, and nodal efficiency derived from 50 nodes in the F-MS and NF-MS groups were not different from those of the HCs. However, relative to the NF-MS group, the F-MS group exhibited reduced nodal efficiency and nodal betweenness centrality in the right mediodorsal medial magnocellular nuclei (all with FDR corrected *p* < 0.05, Figure 3). The remaining nodes were not different.

### 3.5. Correlation Analysis

The volume of T2 lesions in the patients was negatively correlated with the right lateral geniculate (rho = −0.501, *p* = 0.001), right pulvinar anterior (rho = −0.570, *p* < 0.001), right pulvinar medial (rho = −0.595, *p* < 0.001), left pulvinar inferior (rho = −0.541, *p* < 0.001), and right pulvinar inferior nuclei (rho = −0.582, *p* < 0.001). Interestingly, these nuclei all form part of the posterior thalamus. We detected no statistically significant correlations between the FSS score and thalamic nuclei volumes after multiple corrections. Furthermore, the disease duration was negatively correlated with the right pulvinar inferior (rho = −0.323, *p* = 0.045), right lateral geniculate (rho = −0.321, *p* = 0.045), right ventromedial (rho = −0.427, *p* = 0.007), right ventral posterolateral (rho = −0.351, *p* = 0.029), right parafascicular (rho = −0.428, *p* = 0.007), right centromedian (rho = −0.421, *p* = 0.009), and right paratenial nuclei (rho = −0.362, *p* = 0.023) without multiple corrections. The patients’ ages were negatively correlated with the right pulvinar inferior nucleus (rho = −0.329, *p* = 0.041) without multiple corrections.

## 4. Discussion

Although we did not identify differences in the volumes of the whole thalamus and the individual thalamic nuclei in the F-MS patients as compared to the NF-MS patients, our results demonstrated that right anteroventral, right central lateral, right lateral geniculate, right pulvinar anterior, left pulvinar medial, and left pulvinar inferior nuclei atrophy was observed only in F-MS patients. Furthermore, the F-MS patients exhibited a significantly different intrinsic thalamic network from the NF-MS patients. The F-MS patients had significantly increased *Lp* and decreased nodal efficiency and nodal betweenness centrality of the right mediodorsal medial magnocellular nucleus.

The thalamic nuclei volumes were significantly reduced even in patients who demonstrated no indication of thalamic degeneration by visually inspecting conventional MRI scans. The correlation analysis demonstrated that the volume of only four nuclei significantly correlated with the volume of WM lesions among the numerous atrophied thalamic nuclei in the patients. Deppe et al. concluded that PwMS with low WM lesion load and no thalamic lesions already had reduced average thalamic volume [30]. Therefore, we demonstrated that thalamic degeneration and WM lesions cannot fully explain the pathological substrates of thalamic volume loss. While we identified atrophy, we did not identify correlation between the atrophied thalamic nuclei volumes and fatigue severity. This was supported by a longitudinal study [31] that reported no relationship between fatigue severity and brain atrophy initially but observed it after six years of follow-up.

In the present study, right pulvinar inferior nucleus atrophy was identified only in the F-MS patients and worsened with age and disease duration. In contrast to the NF-MS patients, the F-MS patients had a greater average age (33 vs. 29.5 years) and longer disease duration (2.5 vs. 1.7 years). This indicates that the atrophy of the right pulvinar inferior nucleus in the F-MS is probably attributable to the increased age and longer disease duration and is not solely due to fatigue. Furthermore, disease duration negatively related with the with the volume of multiple atrophied thalamic nuclei located in the right hemisphere, albeit this was uncorrected for multiple comparisons. Several studies confirmed that the annual rate of thalamic atrophy in the early stages is approximately 1.0% [15,32]. Consequently, disease duration plays an important role in thalamic atrophy. In addition, our results suggest that most of the atrophied thalamic nuclei in PwMS are located in the right hemisphere, indicating right lateralization of thalamic atrophy in PwMS, which may be related to the dominance of fiber tracts of the right side at thalamic level [33].

The anteroventral nucleus has the most extensive interactions with the subiculum and retrosplenial cortex, and fires rhythmically and synchronously with the hippocampal theta (4–7 Hz) frequency [34]. Therefore, it is a key component of the Papez circuit, which is related to and mnemonic functions in the brain [35]. In the present study, we determined that the F-MS patients had decreased anteroventral nucleus volume. PwMS demonstrated comparable performance regarding tasks of working memory with limited processing speed demands, but their performance declined in contrast to that of HCs when processing speed demands become more salient [36]. Furthermore, the PwMS needed more repetition or learning trials to master new information than HCs. DeLuca et al. used a task-based paradigm to describe increased cerebral activation within the thalamus during cognitive performance or cognitive fatigue over time [37]. Their observations suggested that fatigued PwMS expend additional effort to perform a task adequately. Based on this, we may speculate that the reduced anteroventral nucleus volume or the resulting functional compensation may lead to MS-related cognitive fatigue.

The central lateral nucleus is considered a part of the intralaminar thalamic nuclear group. The privileged role of the central lateral nucleus in forebrain arousal and cortico-cortical communication control has made it a target for clinical deep brain stimulation of the central thalamus in a minimally conscious state [38]. Moreover, central lateral nucleus and medial dorsal tegmental tract co-activation significantly affected behavioral facilitation [39]. Experienced as a lack of mental and physical resources, fatigue in PwMS could mainly be driven by hyperstable arousal regulation, which is expected to prematurely exhaust PwMS [40]. Our results demonstrated that the central lateral nucleus volume decreased in fatigued patients. Therefore, we tended toward the hypothesis that the changes in arousal regulation patterns and behavioral facilitation related to the central lateral nucleus could also be related to the pathophysiological mechanism of fatigue in PwMS.

A widely distributed network of regions in the frontal and parietal cortices is considered to govern the attentional selection of behaviorally relevant information. This high-order attention network has a cortical pathway to the lateral geniculate nucleus via V1 and a subcortical pathway to the lateral geniculate nucleus via the thalamic reticular nucleus [41]. Instead of merely strengthening a selected object, attention regulates local trade-offs in processing capability so that enhancing a single object occurs at the cost of the immediate surroundings. This inhibition of attention activity derives from the thalamus, a mechanism that facilitates signal processing with behavioral importance [42]. Attention deficits are one of the most frequently mentioned difficulties among PwMS. The specific issue is the limited capacity to maintain informational bits in one’s focus while manipulating them [43]. In the context of a distracting environment, even patients with mild MS can experience minor cognitive processing abnormalities [44]. Higher performance fatigability on the Continuous Performance Test, a well-known automated measure of sustained attention, was associated with greater self-reported physical and cognitive fatigue in PwRRMS [45]. Therefore, we speculated that alterations in the higher-order attentional networks resulting from reduced lateral geniculate nucleus volume may contribute to the development of MS-related fatigue.

The pulvinar nucleus is traditionally subdivided into the medial, lateral, inferior, and anterior nuclei [46]. Our results demonstrated that the pulvinar anterior nucleus was atrophied only in F-MS patients. It projected towards the somatosensory cortex, which is compatible with a tight coupling with the sensorimotor regions [47]. Based on its association with networks implicated in cognition, this subregion may have motor selection and programming roles [48,49]. Volumetric investigations documented the atrophy of the sensorimotor cortices in fatigued PwMS [50,51], and functional MRI and neurophysiologic investigations demonstrated sensorimotor network dysfunction in fatigued patients during motor tasks [52] and at rest [53]. Moreover, Cogliati Dezza et al. reported that MS-related fatigue increased with functional imbalance between homologous sensorimotor area [54]. The pulvinar anterior nucleus as the primary node of the sensorimotor network may be early atrophied, thereby becoming a central mechanism of MS-related fatigue.

We also demonstrated via graph theoretical analysis that the F-MS patients had different intrinsic thalamic global and regional networks from the NF-MS patients. Despite the common small-world topology, the F-MS patients had significantly increased *Lp* compared to the NF-MS patients. The NF-MS patients’ results were intermediate between that of the F-MS and HCs. The *Lp* calculates the average distance or routing efficiency between any two nodes in a network, where lower values indicate better routing efficiency [55]. Accordingly, the fundamental organizational principle it supports is functional integration in the brain. Our results demonstrated that the PwMS had poor global functional integration of the intrinsic thalamic network, and this decrease was more noticeable in the F-MS patients. The existence of more atrophied thalamic nuclei in the F-MS patients may explain the uneven degree of decreased functional integration in PwMS. Few studies have investigated *Lp* changes in PwMS. A recent study on cortical structural connections reported that greater path length correlated with average cognition when PwMS were compared with HCs, indicating that MS with cognitive impairment exhibits more random network features and lower global efficiency [56]. We discovered new evidence that may aid the understanding of why fatigued PwMS need to exert greater effort to preserve cognitive functioning. Moreover, the F-MS patients demonstrated less nodal efficiency and betweenness centrality of the right mediodorsal medial magnocellular nucleus than the NF-MS patients. Nodal betweenness centrality measures the influence of a node over information flow between other network nodes while nodal efficiency characterizes parallel information transfer effectiveness. The reductions of these metrics in a thalamic subregion imply the disruption of interconnectivity with other subregions in the intrinsic thalamic network. However, no studies have focused on the intrinsic thalamic network. Our findings revealed that the F-MS patients had mediodorsal medial magnocellular nucleus dysfunction in integrating and processing information even though its volume was unaltered.

Although this is the first research to investigate the volumes of various thalamic nuclei and the intrinsic network alterations in fatigued PwMS, it has several limitations. First, this was a cross-sectional study with a small sample size. This design rendered it challenging to find the causal link between structural alterations and clinical characteristics. While we observed atrophy, we could not confirm the causal relationship between it and the exact pathological mechanisms of MS-related fatigue. Longitudinal research with larger sampling sizes is required to validate our findings. Second, we used the MoCA to assess the cognitive abilities of the PwMS rather than more sensitive tests, such as the Symbol Digit Modalities Test (SDMT), Verbal Learning Test–Second Edition, and the Brief Visuospatial Memory Test. Recent data indicated significant differences in the MoCA (*p* = 0.016) and SDMT (*p* < 0.001) between PwRRMS (n = 48) and HCs (n = 26) [57]. The different sensitivity of these two tests may result in the non-detection of some patients with mild cognitive impairment, which in turn may have affected the reliability of our results. In the future, we will use more specialized and sensitive tests used for evaluating psychological and cognitive abilities. Third, previous studies identified pain as a factor contributing to gray matter reduction [18]. The most prevalent condition among the MS cases over the five-year period before a first demyelinating event was pain [58]. The lack of patient pain ratings in our study may also have affected the reliability of our experimental results. Awareness of this issue will aid more careful design in future experiments. Fourth, the thalamic volume may differ between human males and females, and regression was performed for this factor in network analysis. However, in order to improve the credibility of the experiment, we must perform some fine-grained and subgroup analyses in the future with larger samples. Lastly, we investigated the intrinsic thalamic network using a graph-theoretic method based on the thalamic nuclei volume. With this approach, we could only calculate the network metrics at the group level instead of individual level. Consequently, performing a correlation study between network metrics and clinical factors was challenging.

## 5. Conclusions

Fatigued PwMS exhibited more atrophied thalamic nuclei, poorer intrinsic thalamic network global functional integration, and disrupted nodal right mediodorsal medial magnocellular nucleus interconnectivity with other intrinsic thalamic network subregions. These findings might aid the clarification of the underlying pathogenesis of MS-related fatigue.

## Figures and Tables

**Table 1:** Demographic, clinical, and cerebral imaging characteristics in the F-MS, NF-MS, and HCs groups.

		F-MS	NF-MS	HCs	*p* Value	*p* Value	*p* Value	*p* Value
					All Groups	F-MS vs. HCs	NF-MS vs. HCs	F-MS vs. NF-MS
N		25	25	40	-	-	-	-
Female/male		16/9	17/8	26/14	0.952 ^a^	0.935 ^a^	0.804 ^a^	0.692 ^a^
OB (+)/OB (-)		11/14	15/10	-	-	-	-	0.258 ^a^
Age (y)	Median (IQR)	33 (25.5)	29.5 (24)	28 (25)	0.242 ^b^	-	-	0.247 ^c^
Education (y)	Median (IQR)	12 (9)	14(11.2)	13 (9)	0.183 ^b^	-	-	0.152 ^c^
EDSS	Median (IQR)	2.5 (1)	2 (1.5)	-	-	-	-	0.600 ^c^
MoCA	Median (IQR)	27 (26.7)	28 (27)	28 (27)	0.095 ^b^	-	-	0.112 ^c^
BDI-II	Median (IQR)	7.5 (3.7)	4.5 (3)	4 (3)	0.118 ^b^	-	-	0.106 ^c^
FSS	Median (IQR)	4.8(4.2)	2.7 (2.3)	2.3 (1.9)	**0.000 ^b^**	**0.000 ^b^**	0.335 ^b^	**0.000 ^b^**
PSQI	Median (IQR)	6 (5)	5 (2.8)	5 (4)	0.130 ^b^	-	-	0.147 ^c^
Lesion volume (mL)	Median (IQR)	6.3 (2.6)	6.4 (2.1)	-	-	-	-	0.381 ^c^
Disease duration (y)	Median (IQR)	2.5 (1.5)	1.7 (1.4)	-	-	-	-	0.619 ^c^
CortVol (mL)	Mean (SD)	470.05 ± 38.87	463.43 ± 58.04	489.64 ± 47.32	0.102 ^d^	0.376 ^d^	0.169 ^d^	1.000 ^d^
SubCortGrayVol (mL)	Mean (SD)	56.44 ± 7.26	55.79 ± 6.94	61.89 ± 4.76	**0.000 ^d^**	**0.002 ^d^**	**0.001 ^d^**	1.000 ^d^
TotalGrayVol (mL)	Mean (SD)	629.79 ± 51.83	625.43 ± 74.01	660.86 ± 58.62	0.054 ^d^	0.169 ^d^	0.126 ^d^	1.000 ^d^
WhiteVol (mL)	Mean (SD)	446.24 ± 65.72	437.05 ± 62.94	480.01 ± 53.09	**0.017 ^d^**	0.100 ^d^	**0.035 ^d^**	1.000 ^d^
eTIV (mL)	Mean (SD)	1336.74 ± 256.13	1328.01 ± 278.80	1396.01 ± 282.20	0.584 ^d^	1.000 ^d^	1.000 ^d^	1.000 ^d^

F-MS: Patients with multiple sclerosis presenting fatigue; NF-MS: Patients with multiple sclerosis not presenting fatigue; HCs: Healthy controls; SD: Standard deviation; IQR: interquartile range; OB: IgG oligoclonal bands in CSF; EDSS: Expanded Disability Status Scale; MoCA: Montreal Cognitive Assessment; BDI-II: Beck Depression Inventory II; FSS: Fatigue Severity Scale; PSQI: Pittsburgh Sleep Quality Index; CortVol: cortex volume; SubCortGrayVol: subcortical gray matter volume; TotalGrayVol: total gray matter volume; WhiteVol: white matter volume; eTIV: estimated total intracranial volume. Significant values are in boldface. ^a^ Pearson’s chi-square test. ^b^ Kruskal–Wallis test. ^c^ Mann–Whitney U test. ^d^ One-way ANOVA with post hoc Bonferroni’s test.

**Table 2:** Differences in the individual thalamic nuclei volumes among the F-MS, NF-MS, and HC groups.

	F-MS			NF-MS				HCs	
Thalamic Nucleus	Mean, %	SD, %	* p-Value	Mean, %	SD, %	* p-Value	† *p*-Value	Mean, %	SD, %
Whole thalamus	0.9601	0.1853	0.0300	0.9853	0.1689	0.0670	1.000	1.0919	0.1699
Lt. whole thalamus	0.5049	0.0946	0.0157	0.5284	0.1064	0.1426	0.4497	0.5687	0.0924
Rt. whole thalamus	0.4588	0.0893	0.0047	0.4583	0.0782	0.0067	0.9846	0.5232	0.0807
Anterior group									
Lt. anteroventral	0.0086	0.0019	0.0032	0.0090	0.0028	0.0292	0.5585	0.0103	0.0019
Rt. anteroventral	0.0081	0.0016	‡ 0.0001	0.0088	0.0020	0.0172	0.2353	0.0101	0.0020
Lateral group									
Lt. laterodorsal	0.0014	0.0004	‡ 0.0010	0.0013	0.0007	‡ 0.0009	0.8441	0.0020	0.0008
Rt. laterodorsal	0.0012	0.0006	‡ 0.0001	0.0011	0.0005	‡ 0.0001	0.7220	0.0019	0.0006
Lt. lateral posterior	0.0087	0.0022	0.0654	0.0086	0.0022	0.0629	0.9209	0.0097	0.0017
Rt. lateral posterior	0.0079	0.0020	0.0068	0.0079	0.0016	0.0103	0.9925	0.0093	0.0018
Ventral group									
Lt. ventral anterior	0.0301	0.0060	0.0576	0.0306	0.0064	0.1349	0.7800	0.0330	0.0050
Rt. ventral anterior	0.0291	0.0057	0.0327	0.0294	0.0053	0.0650	0.8663	0.0323	0.0052
Lt. ventral anterior magnocellular	0.0024	0.0005	0.0851	0.0025	0.0005	0.4686	0.4170	0.0026	0.0004
Rt. ventral anterior magnocellular	0.0024	0.0005	0.1615	0.0024	0.0004	0.1107	0.8115	0.0026	0.0004
Lt. ventral lateral anterior	0.0478	0.0095	0.1342	0.0493	0.0090	0.4261	0.5769	0.0512	0.0075
Rt. ventral lateral anterior	0.0449	0.0087	0.0901	0.0445	0.0067	0.0787	0.8930	0.0484	0.0073
Lt. ventral lateral posterior	0.0632	0.0133	0.1111	0.0658	0.0115	0.4604	0.4902	0.0681	0.0102
Rt. ventral lateral posterior	0.0585	0.0113	0.0763	0.0581	0.0082	0.0688	0.9018	0.0631	0.0095
Lt. ventral posterolateral	0.0740	0.0152	0.0612	0.0802	0.0151	0.7458	0.1973	0.0815	0.0144
Rt. ventral posterolateral	0.0635	0.0132	0.0506	0.0642	0.0098	0.1029	0.8369	0.0698	0.0119
Lt. ventromedial	0.0020	0.0004	0.0589	0.0021	0.0004	0.4866	0.3271	0.0022	0.0004
Rt. ventromedial	0.0018	0.0005	0.0808	0.0017	0.0004	0.0761	0.9167	0.0020	0.0004
Intralaminar group									
Lt. central medial	0.0047	0.0012	0.0246	0.0048	0.0015	0.0916	0.6818	0.0054	0.0010
Rt. central medial	0.0045	0.0012	0.0082	0.0044	0.0012	0.0059	0.8235	0.0053	0.0010
Lt. central lateral	0.0022	0.0005	0.0013	0.0023	0.0006	0.0117	0.6043	0.0028	0.0006
Rt. central lateral	0.0021	0.0004	‡ 0.0001	0.0022	0.0004	0.0036	0.4318	0.0026	0.0006
Lt. paracentral	0.0003	0.0001	0.0296	0.0003	0.0001	0.1690	0.5310	0.0003	0.0001
Rt. paracentral	0.0003	0.0001	0.0654	0.0003	0.0001	0.0258	0.6612	0.0004	0.0001
Lt. centromedian	0.0189	0.0037	0.0507	0.0194	0.0031	0.1701	0.6618	0.0208	0.0037
Rt. centromedian	0.0177	0.0035	0.0714	0.0174	0.0023	0.0418	0.7686	0.0192	0.0032
Lt. parafasicular	0.0044	0.0011	0.1080	0.0043	0.0010	0.0534	0.7133	0.0048	0.0009
Rt. parafasicular	0.0044	0.0010	0.1996	0.0042	0.0007	0.0283	0.3776	0.0047	0.0008
Medial group									
Lt. paratenial	0.0005	0.0001	0.0369	0.0006	0.0001	0.5393	0.2218	0.0006	0.0001
Rt. paratenial	0.0005	0.0001	0.0144	0.0005	0.0001	0.0140	0.9033	0.0005	0.0001
Lt. reuniens (medial ventral)	0.0009	0.0003	0.0081	0.0009	0.0004	0.0127	0.9802	0.0011	0.0002
Rt. reuniens (medial ventral)	0.0008	0.0003	0.0017	0.0008	0.0003	0.0020	0.9234	0.0010	0.0003
Lt. mediodorsal medial magnocellular	0.0582	0.0120	0.1454	0.0593	0.0189	0.2630	0.8139	0.0639	0.0133
Rt. mediodorsal medial magnocellular	0.0559	0.0129	0.0865	0.0531	0.0132	0.0152	0.4647	0.0616	0.0111
Lt. mediodorsal lateral parvocellular	0.0195	0.0037	0.1365	0.0200	0.0070	0.2917	0.7480	0.0216	0.0050
Rt. mediodorsal lateral parvocellular	0.0189	0.0048	0.0713	0.0177	0.0045	0.0086	0.3995	0.0211	0.0044
Posterior group									
Lt. lateral geniculate	0.0197	0.0057	0.0022	0.0201	0.0051	0.0079	0.8009	0.0238	0.0043
Rt. lateral geniculate	0.0157	0.0046	‡ 0.0002	0.0165	0.0041	0.0035	0.5211	0.0199	0.0034
Lt. medial geniculate	0.0080	0.0016	0.0251	0.0086	0.0018	0.3020	0.3231	0.0093	0.0024
Rt. medial geniculate	0.0085	0.0015	0.1019	0.0086	0.0017	0.1902	0.8267	0.0093	0.0021
Lt. limitans (suprageniculate)	0.0015	0.0005	0.0161	0.0017	0.0005	0.2352	0.3195	0.0018	0.0006
Rt. limitans (suprageniculate)	0.0014	0.0005	0.1960	0.0015	0.0004	0.7682	0.4095	0.0016	0.0005
Lt. pulvinar anterior	0.0164	0.0032	0.0093	0.0178	0.0045	0.2263	0.2520	0.0191	0.0037
Rt. pulvinar anterior	0.0142	0.0030	‡ 0.0009	0.0143	0.0029	0.0022	0.9295	0.0169	0.0029
Lt. pulvinar medial	0.0792	0.0155	‡ 0.0007	0.0845	0.0192	0.0273	0.3295	0.0951	0.0162
Rt. pulvinar medial	0.0698	0.0149	‡ 0.0001	0.0713	0.0164	‡ 0.0003	0.7647	0.0875	0.0146
Lt. pulvinar lateral	0.0144	0.0031	0.2174	0.0155	0.0034	0.9345	0.2697	0.0154	0.0029
Rt. pulvinar lateral	0.0121	0.0033	0.1107	0.0119	0.0022	0.0875	0.8613	0.0132	0.0024
Lt. pulvinar inferior	0.0181	0.0042	‡ 0.0010	0.0190	0.0056	0.0126	0.5421	0.0222	0.0040
Rt. pulvinar inferior	0.0146	0.0036	‡ 0.0001	0.0153	0.0039	‡ 0.0006	0.5958	0.0191	0.0038

SD: Standard deviation; Rt: right; Lt: left. * *p*-value: Comparison with HCs. † *p*-value: Comparison between F-MS and NF-MS subtypes. ‡ *p* < 0.001.

**Table 3:** Differences in the intrinsic thalamic global network in the F-MS, NF-MS, and HC groups.

	**F-MS**		**HCs**				
	**Average Value**	**AUC**	**Average Value**	**AUC**	**Difference**	**Difference in AUC**	***P^AUC^***
*Lp*	2.9883	1.0321	2.4339	0.8419	0.5544	0.1902	* 0.013
*E_glob_*	0.3977	0.1393	0.4589	0.1610	−0.0612	−0.0217	0.1210
*E_loc_*	0.6230	0.2191	0.6410	0.2251	−0.0180	−0.0060	0.6150
*Cp*	0.5351	0.1881	0.5270	0.1848	0.0081	0.0033	0.7240
*σ*	1.3939	0.4859	1.4883	0.5105	−0.0944	−0.0245	0.6990
	**NF-MS**		**HCs**				
	**Average Value**	**AUC**	**Average Value**	**AUC**	**Difference**	**Difference in AUC**	***P^AUC^***
*Lp*	2.4569	0.8377	2.4384	0.8422	0.0185	−0.0045	0.9280
*E_glob_*	0.4746	0.1669	0.4611	0.1619	0.0135	0.0050	0.5660
*E_loc_*	0.6738	0.2367	0.6401	0.2247	0.0337	0.0120	0.3470
*Cp*	0.5624	0.1974	0.5244	0.1839	0.0380	0.0136	0.1710
*σ*	1.4697	0.5128	1.4978	0.5135	−0.0281	−0.0007	0.9880
	**F-MS**		**NF-MS**				
	**Average Value**	**AUC**	**Average Value**	**AUC**	**Difference**	**Difference in AUC**	***P^AUC^***
*Lp*	2.9674	1.0190	2.4411	0.8356	0.5263	0.1834	* 0.038
*E_glob_*	0.4067	0.1426	0.4724	0.1662	−0.0657	−0.0235	0.0680
*E_loc_*	0.6304	0.2218	0.6623	0.2330	−0.0319	−0.0112	0.4140
*Cp*	0.5435	0.1913	0.5523	0.1942	−0.0088	−0.0029	0.8150
*σ*	1.4010	0.4905	1.5359	0.5349	−0.1349	−0.0443	0.5780

AUC: Area under the curve; *Lp*: characteristic path length; *E_glob_*: global efficiency; *E_loc_*: local efficiency; *Cp*: clustering coefficient; *σ*: small-world index. * *p* < 0.05.

## Data Availability

The code in this study is available on request from the corresponding author. The data are not publicly available due to local ethical regulations.
